# Supplementary material for: The ion channel ppk301 controls freshwater egg-laying in the mosquito Aedes aegypti
Source: eLife. 2019 May 21;8:e43963. doi: 10.7554/eLife.43963 (PMC6597239; doi:10.7554/eLife.43963)
Supplement: Transparent reporting form [file elife-43963-transrepform.docx]

***eLife’s* transparent reporting form**

We encourage authors to provide detailed information *within their submission* to facilitate the interpretation and replication of experiments. Authors can upload supporting documentation to indicate the use of appropriate reporting guidelines for health-related research (see [EQUATOR Network](http://www.equator-network.org/%20)), life science research (see the [BioSharing Information Resource](https://biosharing.org/)), or the [ARRIVE guidelines](http://www.plosbiology.org/article/info:doi/10.1371/journal.pbio.1000412) for reporting work involving animal research. Where applicable, authors should refer to any relevant reporting standards documents in this form.

If you have any questions, please consult our Journal Policies and/or contact us: [editorial@elifesciences.org](mailto:editorial@elifesciences.org).

**Sample-size estimation**

- You should state whether an appropriate sample size was computed when the study was being designed
- You should state the statistical method of sample size computation and any required assumptions
- If no explicit power analysis was used, you should describe how you decided what sample (replicate) size (number) to use

Please outline where this information can be found within the submission (e.g., sections or figure legends), or explain why this information doesn’t apply to your submission:

In Figure 1c, the number of experiments run was set at 5 empirically. For Figure 1d-g , Figure 3a-j and 6c,d, sample sizes for behavioral data were determined based on common practice in similar behavior experiments and were limited by the number of animals that could be blood-fed by a human at a given time to generate gravid animals for experimentation. For Figure 3d-j, 6c,d and 7a, where individual animals were assayed, the number was limited by the ability to multiplex each assay and the availability of human labor. In general, we strove for sample sizes that were comparable to other similar data sets generated in the literature. For Figure 4 and Figure 4 – figure supplement 1, enough structures were visualized to be sure that all expression patterns were consistent across animals. In Figure 5 and Figure 6a-b, data collection was limited by the low throughput nature of the technique. Because this was the first functional imaging experiment in the *Aedes aegypti* ventral nerve cord, we had no precedent for acceptable sample size, but used standards from other organisms. Whenever comparing mutant and wild-type animals, sample size was kept consistent across all genotypes throughout experiments.

**Replicates**

- You should report how often each experiment was performed
- You should include a definition of biological versus technical replication
- The data obtained should be provided and sufficient information should be provided to indicate the number of independent biological and/or technical replicates
- If you encountered any outliers, you should describe how these were handled
- Criteria for exclusion/inclusion of data should be clearly stated
- High-throughput sequence data should be uploaded before submission, with a private link for reviewers provided (these are available from both GEO and ArrayExpress)

Please outline where this information can be found within the submission (e.g., sections or figure legends), or explain why this information doesn’t apply to your submission:

All replicate information is found in the figure legends and Supplementary raw data file

**Statistical reporting**

- Statistical analysis methods should be described and justified
- Raw data should be presented in figures whenever informative to do so (typically when N per group is less than 10)
- For each experiment, you should identify the statistical tests used, exact values of N, definitions of center, methods of multiple test correction, and dispersion and precision measures (e.g., mean, median, SD, SEM, confidence intervals; and, for the major substantive results, a measure of effect size (e.g., Pearson's r, Cohen's d)
- Report exact p-values wherever possible alongside the summary statistics and 95% confidence intervals. These should be reported for all key questions and not only when the p-value is less than 0.05.

Please outline where this information can be found within the submission (e.g., sections or figure legends), or explain why this information doesn’t apply to your submission:

All statistical tests are indicated in the figure legends. Where possible we show the data as dot plots or box plots to maximize information content. All raw data are available for scrutiny by reviewers and eventual readers, allowing the re-plotting or re-analysis of our data if desired.

(For large datasets, or papers with a very large number of statistical tests, you may upload a single table file with tests, Ns, etc., with reference to sections in the manuscript.)

**Group allocation**

- Indicate how samples were allocated into experimental groups (in the case of clinical studies, please specify allocation to treatment method); if randomization was used, please also state if restricted randomization was applied
- Indicate if masking was used during group allocation, data collection and/or data analysis

Please outline where this information can be found within the submission (e.g., sections or figure legends), or explain why this information doesn’t apply to your submission:

For all behavior and imaging, animals were hatched and reared alongside each other in the same humidified tropical room. For population behavior assays in Figure1, where only wild-type animals were used, animals were reared together and separated at random into groups after blood-feeding. In Figure 1e,g, animals were binned into evenly sized groups selected at random for each concentration of the survival curve. For individual animal tracking experiments in Figure 3d and e, the side of each solution within the assay was alternated to avoid side bias, and genotype position was varied randomly among different trays on different days. Experiments in Figure 3, Figure 6c,d, and 7a were performed or scored anonymized to genotype or solute identity. For imaging experiments, animals expressing the GCaMP6s were sorted for fluorescence as larvae and sexed as pupae, at which point they were separated randomly into cages containing ~10 male and female animals that were to be used for imaging on a single day. These were blood-fed 4 days prior to imaging, and all animals that were not used were discarded. Behavior was anonymized when possible. All experiments in Figure 3d-j and Figure 6c,d were anonymized by genotype or solute identity. The videos in Figure 7a were scored anonymized to genotype.

**Additional data files (“source data”)**

- We encourage you to upload relevant additional data files, such as numerical data that are represented as a graph in a figure, or as a summary table
- Where provided, these should be in the most useful format, and they can be uploaded as “Source data” files linked to a main figure or table
- Include model definition files including the full list of parameters used
- Include code used for data analysis (e.g., R, MatLab)
- Avoid stating that data files are “available upon request”

Please indicate the figures or tables for which source data files have been provided:

All raw data in the paper are available in the uploaded Supplementary File 1 (.xlsx)
